# Older Person’s Participation in Life-Enhancement Activities: A Mixed-Methods Observational Study

**DOI:** 10.1093/geroni/igaf122.3033

**Published:** 2025-12-31

**Authors:** Amarjot Gill, Sharon Hewner, Jihnhee Yu, Tania Von Visger

**Affiliations:** University at Buffalo, Buffalo, New York, United States; University at Buffalo, New York, United States; University at Buffalo, Buffalo, New York, United States; University at Buffalo, Buffalo, New York, United States

## Abstract

This study examines older persons’ experiences and participation in life-enhancement activities in a Canadian long-term care (LTC) facility. Naturalistic observations of 20 life-enhancement activity sessions were conducted in a single LTC facility that includes 111 older persons in September 2024. Data were collected through field notes and guiding questions for systematic observation. NVivo software (version 1.7.1) was used for qualitative thematic analysis, and SPSS software (version 30.0) was used for quantitative analysis. We used Kruskal-Wallis and Mann-Whitney U tests for analysis, with results considered significant if p < 0.05. There were significant differences in engagement levels: self-initiative (p < 0.001), assistance-seeking frequency (p = 0.002), and social interaction frequency (p < 0.001) across activities. Social interaction frequency also significantly differed by mobility status (wheelchair, walker, independent) (p = 0.014). Interpersonal conflict incidents (p < 0.001) and positive emotional expressions (p < 0.001) varied across activity locations (TV room versus Activity Room). Participants displayed significantly more distractions in the TV Room than in the Activity Room (p = 0.001). Four themes emerged from thematic analysis: 1) participation barriers, 2) activity contextual factors, 3) facilitator support strategies, and 4) social interactions and emotional well-being. Individual (mobility) and situational (activity location) factors can influence older persons’ engagement in life-enhancement activities. For older persons to achieve active participation in life-enhancement activities, we must consider their mobility needs, a non-disruptive environment, and facilitator support. Life enhancement activities benefit from facilitators promoting independence and teamwork, improving participation and social connections.

